# Dynamic Multi-Phase-Fused Photon-Counting CT for Pre-TACE Feeding Artery Mapping and DSA Procedural Road-Mapping in Hepatocellular Carcinoma

**DOI:** 10.3390/cancers18193152

**Published:** 2026-09-29

**Authors:** Hao Chen, Hao Deng, Xin Wei, Xi Liu, Weijuan Chen

**Affiliations:** Department of Radiology, The Second Affiliated Hospital of Chongqing Medical University, Chongqing 400010, China; 306425@hospital.cqmu.edu.cn (H.C.); 302266@hospital.cqmu.edu.cn (H.D.); weixin@hospital.cqmu.edu.cn (X.W.)

**Keywords:** photon-counting CT, hepatocellular carcinoma, transarterial chemoembolization, target vessel, digital subtraction angiography, pathway planning

## Abstract

**Background/Objectives:** Accurate preoperative feeding artery mapping remains challenging for hepatocellular carcinoma (HCC) even with modern CT techniques. This study aimed to optimize dynamic multi-phase photon-counting CT (PCCT) hepatic artery protocols and assess dynamic-fusion PCCT against best single-phase PCCT and energy-integrating-detector dual-energy CT (EID-DECT) for feeding artery detection, vascular-map construction and digital subtraction angiography (DSA) path-planning prior to transarterial chemoembolization (TACE) in HCC. **Methods:** This prospective single-center study contained workflow-optimization among cirrhotic non-HCC patients and clinical validation in TACE-candidate HCC patients. Two radiologists performed blinded independent reading. The composite reference standard consisted of DSA, selective/superselective angiography, available cone-beam computed tomography (CBCT) and embolization records. Primary endpoint: patient-level paired difference in DSA-verified feeding artery coverage. Secondary endpoints included complete-atlas rate, DSA-path-prediction accuracy, missed-vessel count and extrahepatic-supply detection. A pre-specified comparison between dynamic-fusion PCCT and EID-DECT was performed. **Results:** Dynamic phase 1–6 fusion was the optimal PCCT scheme, while optimal single-phase varied individually. Dynamic-fusion PCCT achieved superior feeding artery coverage (97.78% ± 8.61% vs. 88.33% ± 15.37%, difference 9.44 pp, 95% CI 1.82–17.06, *p* = 0.016), complete-atlas rate (93.3% vs. 53.3%, *p* = 0.039) and path-prediction accuracy (96.67% ± 11.18% vs. 80.00% ± 24.49%), with fewer missed culprit vessels. Two extrahepatic feeders were only detected by dynamic-fusion PCCT. Compared with EID-DECT, dynamic-fusion PCCT showed favorable atlas-related metrics without statistically significant difference in feeding artery coverage. **Conclusions:** Dynamic-fusion PCCT outperforms single-phase PCCT in pre-TACE feeding artery mapping and DSA path-prediction and shows potential benefits over EID-DECT. It delivers comprehensive preoperative vascular data and may reduce intraoperative exploratory procedures.

## Simple Summary

Transarterial chemoembolization is a key local treatment for unresectable hepatocellular carcinoma. Complete preoperative identification of tumor-feeding arteries helps operators plan interventional pathways and reduce exploratory procedures during surgery. This prospective study optimized a dynamic multi-phase fused photon-counting CT workflow and compared its performance with single-phase photon-counting CT and conventional dual-energy CT. Our results show that fused images improve the detection of feeding arteries, the completeness of vascular maps and prediction of interventional pathways. This imaging technique may supply more comprehensive preoperative vascular information, which has potential to streamline interventional workflows for hepatocellular carcinoma treatment.

## 1. Introduction

Transarterial chemoembolization (TACE) is a key locoregional therapy for unresectable hepatocellular carcinoma (HCC). As treatment strategies and interventional tools have advanced, practice has shifted from lobar or segmental embolization toward selective and superselective approaches. Consequently, operators must not only localize tumor anatomy and vascular supply but also delineate the origin and branching hierarchy of culprit vessels, their continuous course to the tumor, and nearby important non-target branches before the procedure. Failure to identify culprit vessels can result in residual tumor and incomplete embolization, while incorrect path assessment or missed extrahepatic supply can increase ineffective angiography and provoke vascular spasm from repeated superselective catheterizations [1,2,3].

Digital subtraction angiography (DSA) remains the primary method for confirming tumor staining and identifying culprit vessels during TACE. However, its two-dimensional projection images are vulnerable to vessel overlap and projection-angle artifacts. In addition, under current clinical practice, pre-TACE planning mainly relies on routine contrast-enhanced CT or MRI acquired 1–4 weeks prior to the procedure in accordance with clinical guidelines. Conventional single-phase cross-sectional imaging can delineate gross tumor morphology, yet frequently fails to demonstrate the complete course of small-caliber tumor-feeding arteries. This may lead operators to focus primarily on lesion localization while overlooking feeding artery anatomy. There remains an unmet clinical demand for preoperative CT techniques capable of comprehensive feeding artery mapping to support superselective TACE [2]. Recently, intraoperative cone-beam computed tomography (CBCT), dual-phase CBCT navigation, and automatic feeding artery detection software have been introduced to complement DSA. Studies indicate that three-dimensional feeding artery maps improve feeding artery detection and reader consistency, while reducing exploratory angiography, shortening fluoroscopy time, and facilitating superselective treatment [4,5,6]. Most of these approaches, however, require intra-arterial contrast injections after catheter placement and therefore constitute workflow optimizations confined to the interventional suite. Their effectiveness can also be compromised by respiratory motion during the procedure.

Multi-temporal information integration has likewise been validated in Angio-CT. After Takada et al. performed three-dimensional vascular analysis with Angio-MDCT, the numbers of DSA and CT examinations required for TACE decreased significantly, and detection of extrahepatic blood supply improved [7]. Ohta et al. acquired CT images in the early and late arterial phases during preinterventional angiography and, when needed, superimposed the two phases. The feeding artery recognition rate reached 98.0%, indicating that arterial information from different time points is complementary for vascular visualization and tumor staining [8]. Minamiguchi et al. reconstructed an atlas of tumor-feeding arteries using CT during aortography, further supporting that three-dimensional path information aids feeding artery selection [9]. Earlier studies of dual-arterial phase CT similarly suggested that multi-temporal arterial data better visualizes hypervascular lesions and their blood-supply relationships than single-phase information [10].

Photon-counting CT (PCCT) offers a technical foundation for extending this workflow into the preoperative setting. PCCT delivers higher spatial resolution and lower electronic noise and supports conventional multi-energy reconstruction. Low-keV virtual monoenergetic images boost iodine contrast, and ultra-thin slices improve visualization of small visceral arteries [11,12,13]. A prospective CT angiography (CTA) study of visceral arteries found that although 0.2 mm ultra-high-resolution PCCT does not necessarily yield the highest signal-to-noise ratio (SNR) or contrast-to-noise ratio (CNR), it outperforms thicker-slice reconstructions in depicting small arteries, indicating its potential for mapping culprit vessels [14]. Moreover, continuous multi-arterial phase acquisition allows registration and fusion of images across time points, enabling preservation of the vascular purity seen in the early arterial phase while capturing enhanced distal branches and tumors in slightly later phases.

Against this background, we first optimized a multi-phase dynamic arterial acquisition protocol for patients with non-HCC liver cirrhosis and identified the optimal single-phase PCCT, dynamic-fusion PCCT, and predetermined 1–6 phase fusion energy-integrating-detector dual-energy CT (EID-DECT) protocols. Next, for HCC patients scheduled for TACE, we established a composite reference standard comprising DSA, selective/superselective angiography, intraoperative CBCT (when available), and the actual embolization records. We compared single-phase and fusion images in the same PCCT patients and performed prespecified between-group comparisons with the concurrent EID-DECT cohort. Beyond assessing vessel conspicuity, this study evaluates whether the preoperative maps encompass the culprit vessels confirmed by DSA, predict the first-choice DSA access route, and reduce unexpected intraoperative blood supply.

## 2. Materials and Methods

### 2.1. Study Design and Participants

This prospective, single-center controlled study was approved by our hospital’s ethics committee, and all participants provided informed consent. The study was conducted from 11 April 2026, to 10 July 2026, and comprised two parts: image workflow optimization and clinical validation. For image workflow optimization, two parallel cohorts (PCCT and EID-DECT) were established to determine optimized image protocols for each device during multi-phase dynamic acquisition in the arterial phase. For clinical validation, consecutive patients with HCC scheduled for TACE were enrolled into PCCT and EID-DECT cohorts. All patients underwent CT within 7 days before procedure and received DSA/TACE within 7 days, with a maximum allowable interval of 14 days. During this interval, no treatments that could alter tumor blood supply were permitted.

In the image workflow optimization cohort, we included 20 non-HCC cirrhotic patients who underwent PCCT and 20 non-HCC cirrhotic patients who underwent EID-DECT. Inclusion criteria were as follows: age ≥ 18 years; clinically or radiologically confirmed cirrhosis; absence of HCC or other hepatic malignancies; completion of a dynamic contrast-enhanced CT examination on the corresponding scanner; and image quality sufficient for evaluation. Exclusion criteria were prior liver transplantation, liver resection that substantially altered hepatic arterial anatomy, severe respiratory motion artifacts, or incomplete dynamic data.

**Figure 1:**
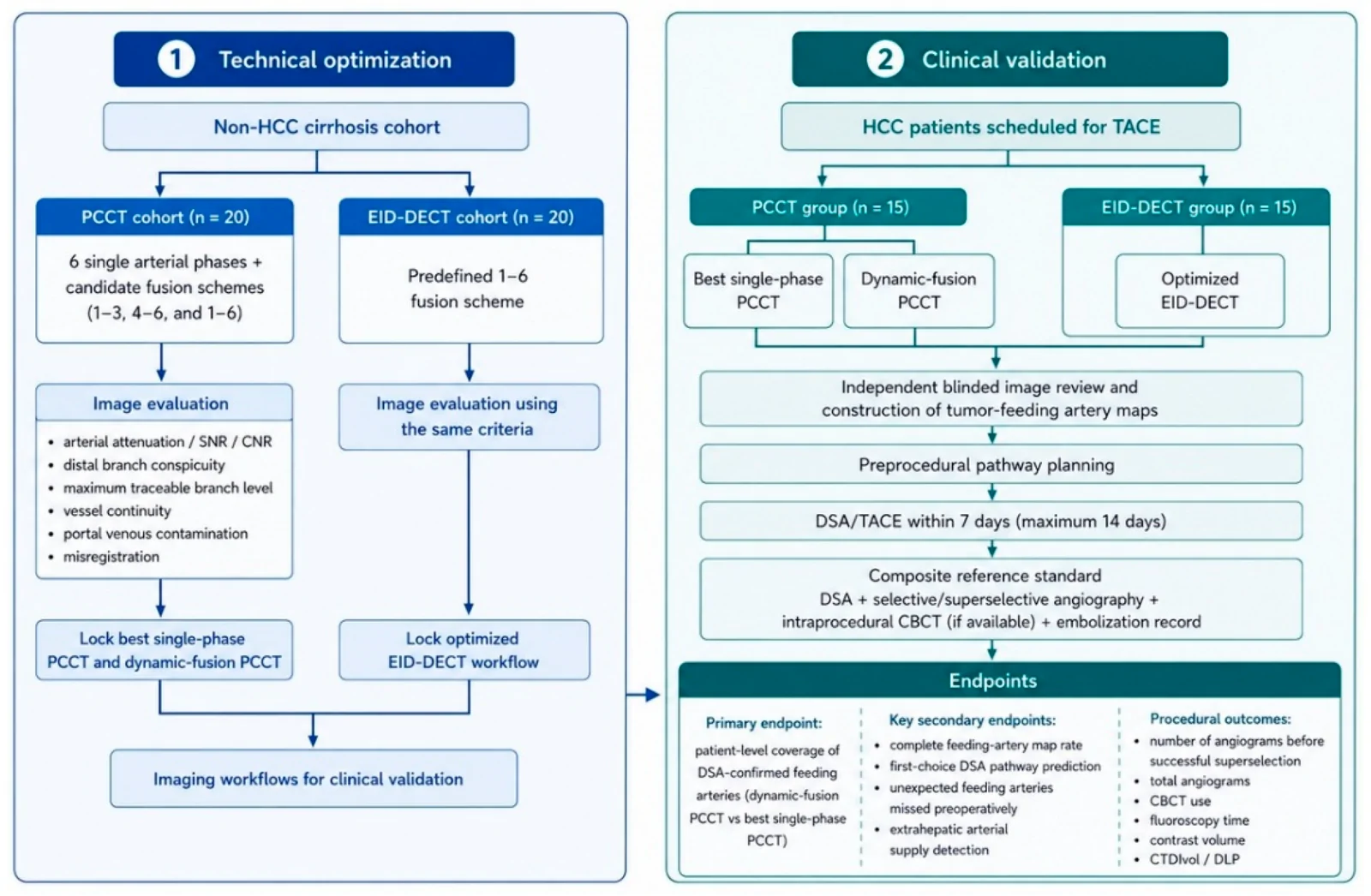
Study design and DSA-based validation workflow.

For the clinical validation, eligible HCC patients were enrolled consecutively during the study period. Inclusion criteria were as follows: a confirmed clinical diagnosis of HCC and planned TACE; completion of dynamic contrast-enhanced PCCT or EID-DECT before the procedure; and complete records of DSA and interventional treatment. Exclusion criteria included prior liver transplantation or hepatectomy that substantially altered hepatic arterial anatomy, diffusely infiltrative HCC, severe arteriovenous fistulas, poor CT image quality, inability to identify the primary responsible vessels by DSA, or major clinical changes between CT and DSA (As shown in Figure 1).

For each patient, a primary target lesion for the current TACE was designated before image review. When multiple lesions were present, priority was given to the lesion most relevant to the current intervention; if priorities were equivalent, the lesion with the largest maximum diameter was selected. Baseline characteristics recorded included the diameter of the largest target lesion, whether lesions were single or multiple, location at the liver apex or in a subcapsular area, Child–Pugh classification, and Barcelona Clinic Liver Cancer (BCLC) stage.

In contrast to previously published studies [11,12,13] that mostly emphasized diagnostic imaging performance of photon-counting CT, this work reports a prospective clinical assessment of a dynamic multi-arterial-phase fusion workflow dedicated to preoperative feeding artery mapping for HCC patients undergoing TACE. A direct head-to-head comparison between dynamic-fusion PCCT and EID-DECT represents the key methodological novelty of our study.

### 2.2. CT Scanning and Image Reconstruction

All PCCT examinations were performed on a Siemens NAEOTOM Alpha scanner (Siemens Healthineers AG, Forchheim, Germany). A total of 65 mL ioversol (350 mg/mL) was intravenously injected at 4 mL/s, followed by a 30 mL saline flush. Continuous whole-liver arterial-phase acquisition started with a 2 s delay once left-atrial attenuation reached 80 HU, generating six arterial phases at 1.5 s intervals. Scan coverage extended from the celiac trunk across the whole liver to include potential inferior phrenic and left-gastric arteries. PCCT parameters: 90 kV, 0.8 mm slice thickness/interval, Br40 kernel, level-4 iterative reconstruction.

EID-DECT examinations were carried out on a Siemens SOMATOM Force (Siemens Healthineers AG, Forchheim, Germany). Apart from scanner-specific acquisition and reconstruction settings, the two groups used identical contrast injection protocols, triggering strategies, dynamic arterial-phase timing, and scan ranges. For EID-DECT, device-specific dual-energy acquisition parameters were applied, and images were reconstructed with a 1.5 mm slice thickness and 1.5 mm interval. The Br40 reconstruction kernel and the vendor’s iterative reconstruction algorithm (intensity level 4) were used. Volume computed tomography dose index (CTDIvol) and Dose-length product (DLP) were recorded for both groups.

PCCT directly discriminates incoming photon-energy bins to enable native multispectral reconstruction without the noise penalty associated with material decomposition. EID-DECT simultaneously acquires two distinct X-ray spectra to support material decomposition and virtual monoenergetic reconstructions. In this study, standard mixed-energy reconstructed images were used for both qualitative and quantitative vascular evaluation; virtual monoenergetic reconstructions were not adopted as primary datasets for image analysis.

We first performed nonrigid registration on the continuous arterial-phase images from PCCT and EID-DECT. Vascular information from different enhancement phases was integrated while preserving native spatial resolution. The post-processing software permitted manual exclusion of individual motion-degraded slices or entire dynamic phases before fusion computation. No slices were excluded in our study, as all patients followed standardized breath-holding instructions during image acquisition. These registered images were then fused on the research workstation (syngo.via VB60S-HF04 (Siemens Healthineers AG, Forchheim, Germany)) according to the preset phase combinations. For each spatial location, the maximum enhancement value across phases was preserved to generate dynamic multi-phase fused images. The fusion algorithm, threshold, and postprocessing parameters were fixed before the formal study and were not altered based on results from study samples. Within each device, identical reconstruction and postprocessing settings were applied across candidate schemes, producing axial images, curved planar reconstructions, maximum intensity projections (MIPs), and inverted-grayscale images.

### 2.3. Strategies for Imaging Protocol Optimization

The PCCT cohort compared six single phases and several predefined fusion schemes (including fusions of phases 1–3, 4–6, and 1–6) within the same patient. Candidate fusion combinations were specified before the formal analysis. Evaluation metrics comprised CT attenuation of the proximal and distal hepatic arteries, SNR, CNR, clarity of subsegmental and smaller branches, maximum traceable branch order, vascular continuity, portal vein contamination, and registration misalignment. The optimal single-phase PCCT and the optimal dynamic-fusion PCCT were selected using the following hierarchical rules: first, any scheme with unacceptable portal vein contamination or registration misalignment was excluded; second, remaining schemes were ranked by continuity and clarity of subsegmental and smaller vessels; when differences were comparable, the scheme with fewer phases and shorter processing time was chosen.

EID-DECT preselects phases 1–6 for dynamic fusion based on prior preliminary experiments and applies the same vascular evaluation criteria as PCCT to assess distal vessel visualization, maximum traceable branches, vessel continuity, portal vein contamination, and registration misalignment.

### 2.4. Clinical Verification

#### 2.4.1. Evaluation of the Responsible Vessel Atlas

The PCCT clinical validation cohort produced two independent image sets per patient: the optimal single-phase PCCT and the optimal dynamic-fusion PCCT. Sequence names and device identifiers were removed from both sets, which were then assigned random numbers. Two abdominal radiologists independently reviewed the images. Reviews were separated by at least 4 weeks, and the readers remained blinded to DSA results and interventional records. The EID-DECT cohort, acquired with the locked optimal protocol, was assessed using the same review criteria. In cases of disagreement, a third senior physician adjudicated; the resulting consensus was used for all statistical analyses.

The image interpreter constructs a structured, responsibility-focused vascular atlas centered on the primary target lesion. For each candidate vessel, the atlas records its origin; intrahepatic versus extrahepatic status; branching level; associated liver lobe or segment; continuity to the tumor margin; vascular integrity; and image clarity. For extrahepatic supply, the origin and course of the right inferior phrenic and adrenal arteries are documented specifically.

To evaluate how well each imaging protocol predicted interventional pathways, radiologists independently recorded, based on their assigned images and blinded to the DSA results, the following: the presumed main culprit vessel, the first-choice vessel for DSA, the number of acute bifurcations (<60°), and their confidence in planning the pathway. These records were used solely for imaging research and did not influence actual interventional decisions. Confidence in pathway planning was rated on a 5-point scale, where 1 indicated an inability to define an implementable pathway and 5 indicated that the culprit vessel and the superselective pathway were clearly identified and an operative plan could be formulated directly.

For objective tumor assessment, regions of interest (ROIs) were placed in three locations: the area of maximal solid enhancement within the target lesion, the adjacent liver parenchyma at the same axial level, and the bilateral psoas major muscles. Background noise was defined as the mean standard deviation (SD) of the bilateral psoas major muscles. Tumor SNR was calculated as the tumor CT value divided by this background noise, and CNR was calculated as the difference between the tumor CT value and the adjacent liver CT value divided by the background noise. These metrics were used to assess visualization of the tumor’s terminal blood-supply region and served as adjunctive indicators supporting angiographic identification of the responsible vessels.

#### 2.4.2. DSA Operation, Composite Reference Standard, and Path Verification

TACE was performed by interventional physicians with at least 5 years of experience. Before each procedure, the operating physician reviewed the available dynamic-fusion PCCT maps or EID-DECT maps and documented a preoperative interventional plan prior to DSA. This plan specified the estimated primary responsible vessels, the preferred first-choice vessels, any potential additional or extrahepatic arterial supplies, and high-risk non-target branches. The optimal single-phase PCCT images were reserved solely for independent image reading and were not used in preoperative path planning; dynamic fusion images informed both research image reading and clinical path planning.

During the procedure, angiography of the celiac trunk or common hepatic artery is performed first, followed by selective and superselective angiography according to the preoperative plan and initial DSA findings; CBCT is used as clinically indicated. The first successfully superselected culprit vessel, the actual operative route, all culprit vessels confirmed by DSA, the number of extrahepatic feeding arteries, the total number of angiographies, whether CBCT was used, fluoroscopy time, and the intraoperative contrast dose were recorded.

After TACE, two interventional physicians independently reviewed all DSA, selective and superselective angiography, CBCT (when available), and the procedural embolization records to establish a composite reference standard. A vessel was considered responsible if it showed tumor staining on selective or superselective angiography corresponding to the target lesion and was corroborated by catheter access, documented embolization extent, or CBCT. Vessels suggested only by preoperative CT but not verified intraoperatively were classified as unverified and excluded from confirmed responsible vessels; vessels confirmed by DSA during the procedure but not identified on the preoperative map were classified as missed responsible vessels. Discrepancies were resolved by a third senior interventional physician.

The accuracy of the imaging-predicted path was defined by whether the first-choice vessel and its upstream branch chain, as identified independently by image readers from the corresponding imaging protocol, matched the actual route of the ultimately successful superselective access to the target vessel on DSA. If the predicted target vessel was correct but a different access route was used because of anatomical constraints, we recorded the correct vessel prediction and the divergent path prediction separately. We also separately recorded the agreement between the preoperative interventional path planned by the operating clinician and the actual DSA route, to assess the clinical value of the preoperative atlas for path planning.

### 2.5. Study Endpoints

The primary endpoint was the paired difference, at the patient level, in the coverage rate of responsible vessels between dynamic fusion images (from independent image reading) and the best single-phase images among the same PCCT patients. Patient-level coverage rate was defined as the number of responsible vessels correctly identified by the preoperative atlas and confirmed by the composite reference standard, divided by the total number of responsible vessels ultimately confirmed by DSA in that patient. Each patient thus yielded a coverage rate for each of the two PCCT image sets. All determinations of responsible vessels and all endpoint analyses were performed on the pre-locked primary target lesions.

Key secondary endpoints included the rate of complete preoperative mapping of culprit vessels, the accuracy of the first DSA path predicted by imaging, and the number of culprit vessels confirmed by DSA that were not identified in the preoperative mapping. Complete mapping was defined as the correct preoperative identification of all culprit vessels confirmed by DSA, with the main culprit vessels continuously traceable from their origins to the tumor periphery and with no DSA-confirmed extrahepatic blood supply missed.

Other secondary endpoints included vessel clarity and continuity, the maximum number of traceable branches, and tumor SNR and CNR. Prespecified intergroup comparisons between dynamic-fusion PCCT and EID-DECT comprised feeding artery coverage rate, rate of complete maps, DSA path-prediction accuracy, visualization of extrahepatic blood supply, and actual interventional operation metrics.

### 2.6. Statistical Analysis

The Shapiro–Wilk test assessed normality of quantitative variables. Continuous variables that were normally distributed were reported as mean ± standard deviation and those that were not were reported as median (interquartile range), M (P_25_–P_75_). Two independent samples were compared using the Mann–Whitney U test. Categorical variables are presented as number of cases (percentage).

#### 2.6.1. Internal Paired Comparison Within PCCT (Dynamically Fused PCCT vs. Optimal Single-Phase PCCT)

For continuous variables, paired *t*-tests were used, and paired differences with 95% confidence intervals (95% CI) were reported. Dichotomous paired outcomes were analyzed with the McNemar test. For each DSA-confirmed culprit vessel, the paired Wilcoxon signed-rank test compared maximum traceable branch level, vascular clarity, and vascular continuity between dynamic-fusion PCCT and the optimal single-phase PCCT. Ordinal grades (1–5 points) are reported as medians with interquartile ranges, M (P_25_–P_75_).

#### 2.6.2. Comparison Between Groups: (Dynamic-Fusion PCCT vs. EID-DECT)

Independent-sample *t*-tests assessed continuous variables, while chi-square tests evaluated categorical variables; Fisher’s exact test was applied when expected cell counts were less than 5. For patient-level coverage rate of responsible vessels, multiple linear regression was used to adjust for prespecified confounders (age, maximum tumor diameter, Child–Pugh liver function classification) and to estimate the adjusted effect size with its 95% CI.

All statistical tests were two-sided, with a significance threshold of α = 0.05. Analyses were performed using SPSS 26.0. *p* values < 0.05 were considered statistically significant.

## 3. Results

### 3.1. Imaging Protocol Optimization

Forty patients with non-HCC cirrhosis were included in the image workflow optimization component, with 20 patients assigned to the PCCT group and 20 to the EID-DECT group. Optimization of the PCCT data indicated that fusing phases 1–6 provided the best dynamic-fusion protocol; the best single phase, however, varied by patient and had to be selected individually based on visualization of the hepatic artery and its distal branches. The dynamic-fusion protocol outperformed the optimal single-phase protocol for clarity of subsegmental and smaller vessels (5 (5, 5) vs. 5 (4, 5), *p* = 0.003), maximum traceable branches (5 (5, 5) vs. 5 (4, 5), *p* = 0.008), and vascular continuity (5 (5, 5) vs. 5 (4, 5), *p* = 0.019). Portal vein contamination and registration misalignment remained within acceptable limits. Informed by these preliminary results, the EID-DECT used a predefined fusion of phases 1–6 and was assessed with the same vascular evaluation criteria applied to PCCT.

### 3.2. Clinical Validation Cohort

Thirty HCC patients were enrolled in the clinical validation: 15 in the PCCT group and 15 in the EID-DECT group. Their demographic, clinical, and baseline tumor characteristics are summarized in Table 1. The DSA composite reference standard identified 36 culprit vessels in the PCCT group and 40 in the EID-DECT group, with a median of 2 culprit vessels per patient in both groups.

#### 3.2.1. Paired Comparison of Dynamic-Fusion PCCT Versus Optimal Single-Phase PCCT

In patients with PCCT, the patient-level DSA-confirmed coverage rate of culprit vessels was 97.78% ± 8.61% on dynamic fusion images versus 88.33% ± 15.37% on the best single-phase images. The paired difference was 9.44 percentage points (95% CI: 1.82–17.06, *p* = 0.016). The rate of complete feeding artery atlases was 93.3% (14/15) for dynamic fusion and 53.3% (8/15) for the best single-phase images, a statistically significant difference (*p* = 0.039).

**Figure 2:**
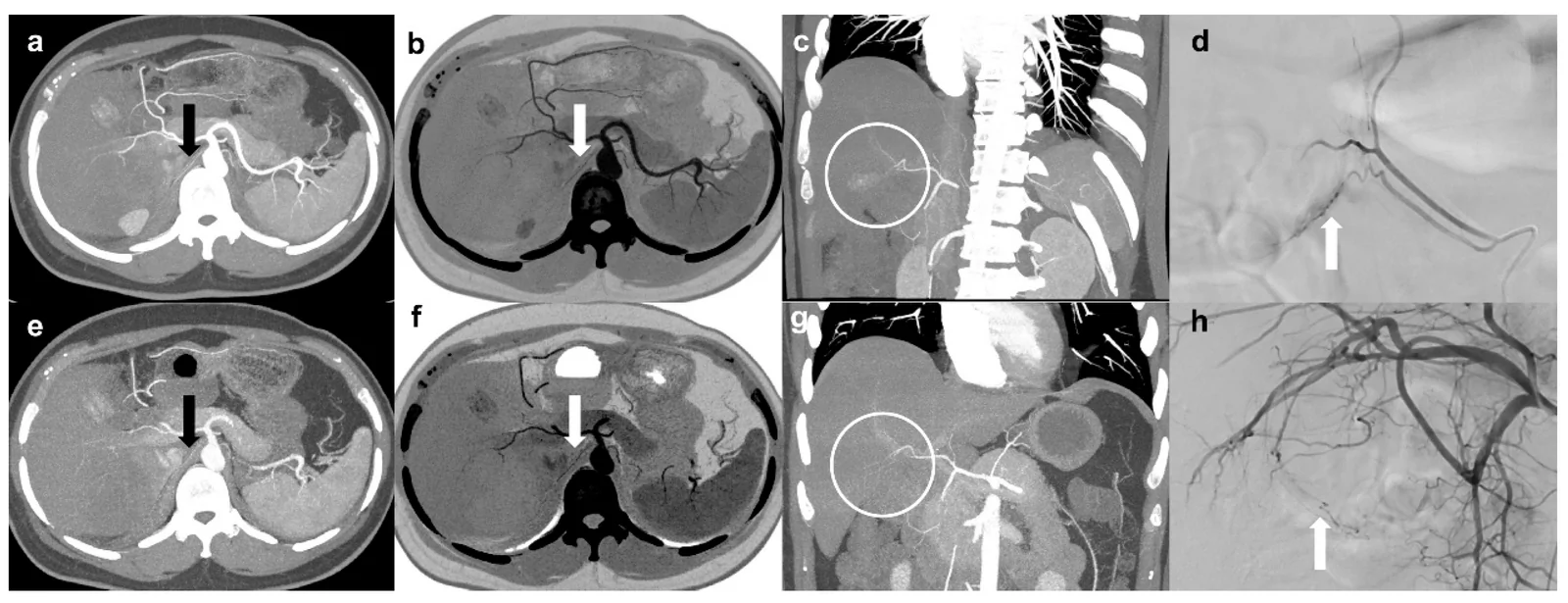
Visualization comparison of tumor feeding arteries for HCC prior to TACE: dynamic multi-phase fused PCCT versus optimal single-phase PCCT. (**a**,**b**) Axial MIP and inverted-grayscale images from dynamic multi-phase fused PCCT clearly depict the course of the right superior adrenal artery (as indicated by the black arrow and white arrow) as an extrahepatic collateral supplying the enhanced portion of the lesion. (**c**) The coronal MIP of the dynamic multi-phase fused PCCT demonstrates lesion enhancement and shows that the hepatic artery (segment S6) contributes to the lesion’s blood supply (as indicated by the white circle). (**e**–**g**) Corresponding axial MIP, inverted-grayscale, and coronal images from the optimal single-phase PCCT of the same patient show poor continuity between the right superior adrenal extrahepatic collateral and the hepatic artery, making it impossible to determine their involvement in lesion perfusion (as indicated by the black arrow, white circle and white arrow). (**d**,**h**) DSA confirms tumor staining and verifies that both the right superior adrenal artery (as shown by the white arrow in d) and hepatic artery segment S6 (as shown by the white arrow in h) contribute to the lesion’s blood supply.

The initial path-prediction accuracy of DSA for dynamically fused images was 96.67% ± 11.18%, compared with 80.00% ± 24.49% for the optimal single-phase images. The number of responsible vessels confirmed by DSA but not identified on preoperative maps was 0.07 ± 0.26 vessels/case for the dynamically fused images and 0.40 ± 0.61 vessels/case for the optimal single-phase images. In the PCCT group, DSA confirmed 2 extrahepatic blood supplies: the dynamically fused images identified both (2/2), whereas the optimal single-phase images identified none (0/2). Because of the small number of positive samples, only descriptive analysis was performed. Tumor SNR and CNR for the dynamically fused images were 14.85 ± 3.22 and 6.82 ± 2.15, respectively, versus 12.36 ± 2.89 and 5.21 ± 1.98 for the optimal single-phase images. The differences in both metrics were statistically significant (*p* = 0.012 and *p* = 0.014, respectively) (as shown in Table 2, Figure 2).

We performed a paired vessel-level analysis of 36 culprit vessels confirmed by DSA in 15 PCCT patients (as shown in Table 3). The maximum traceable branch level was significantly higher with dynamic-fusion PCCT than with optimal single-phase PCCT [median (IQR), 5 (5–5) vs. 5 (4–5); Z = −2.808, *p* = 0.005]. Of the 36 vessels, scores increased for 14 and remained unchanged for 22. Vessel continuity also differed between protocols (Z = −2.000, *p* = 0.046), while vessel clarity did not show a statistically significant difference (Z = −1.732, *p* = 0.083). Overall, both protocols yielded high scores for vessel clarity and continuity, with most ratings clustered near the upper end of the scale.

#### 3.2.2. Comparison of Dynamic-Fusion PCCT Versus EID-DECT

**Figure 3:**
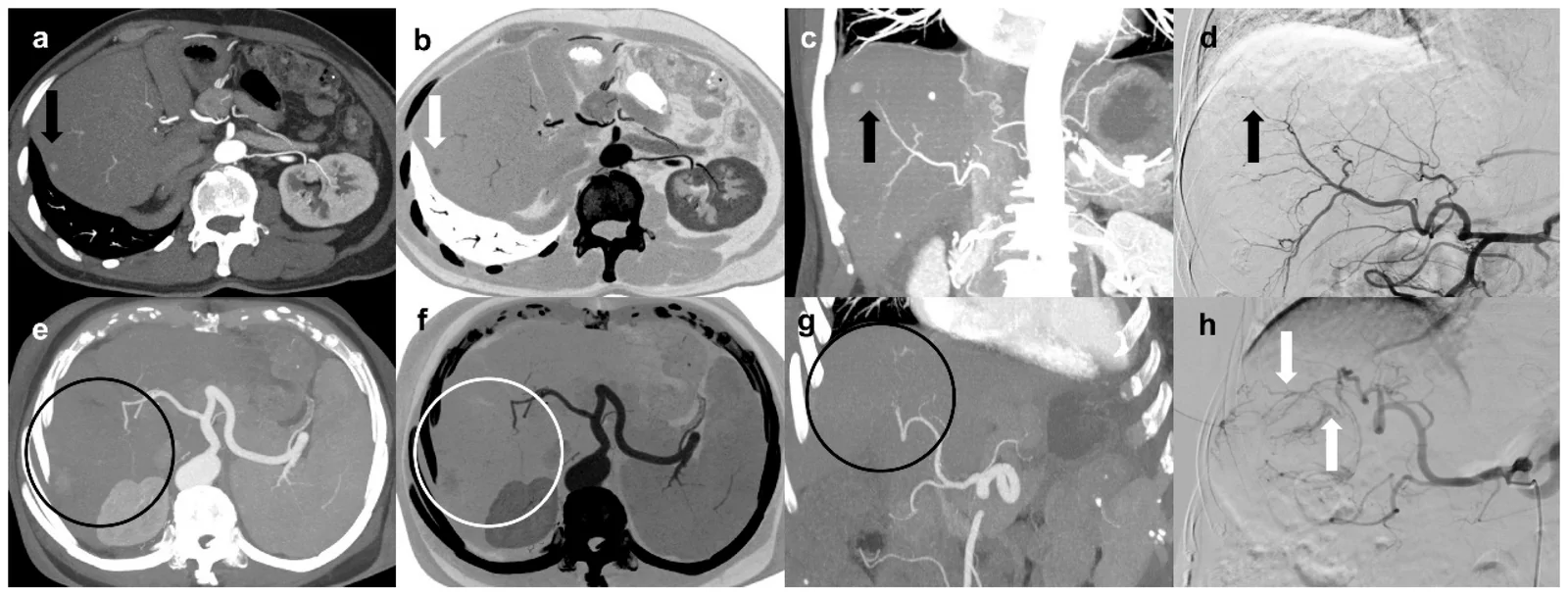
Visualization comparison of tumor feeding arteries for HCC prior to TACE: dynamic multi-phase fused PCCT versus EID-DECT. (**a**,**b**) Dynamic fusion of axial MIP and inverted-grayscale PCCT images faintly reveals fine hepatic artery branches (as indicated by the black arrow and white arrow) extending to the lesion periphery. (**c**) Dynamic fusion of coronal MIP PCCT images demonstrates lesion enhancement and involvement of the hepatic artery (segment S7 as indicated by the black arrow) in its perfusion. (**d**) DSA confirms segment S7 participation in tumor blood supply, showing tumor staining (as indicated by the black arrow). (**e**–**g**) In a second patient, axial MIP, inverted-grayscale, and coronal EID-DECT images do not clearly depict the hepatic artery (as indicated by the black circle and white circle), preventing identification of the vessel responsible for the lesion. (**h**) DSA confirms that hepatic artery segments S5–7 contribute to the lesion’s blood supply (as indicated by the white arrows), with tumor staining.

The patient-level coverage of culprit vessels was 97.78% ± 8.61% in the dynamic-fusion PCCT group and 90.45% ± 12.31% in the EID-DECT group, a difference of 7.33 percentage points (95% CI: −1.01–15.67, *p* = 0.083). The proportion of complete feeding artery maps was 93.3% (14/15) in the PCCT group versus 53.3% (8/15) in the EID-DECT group, a statistically significant difference (*p* = 0.035). The accuracy of predicting the first DSA-selected path was 96.67% ± 11.18% for dynamic-fusion PCCT and 83.33% ± 18.44% for EID-DECT (*p* = 0.032). The mean number of newly identified unexpected culprit vessels per case during the procedure was 0.07 ± 0.26 in the PCCT group and 0.40 ± 0.51 in the EID-DECT group (*p* = 0.040) (as shown in Table 4, Figure 3). In the PCCT group, both extrahepatic blood supplies confirmed by DSA were detected on the dynamic fusion images, with traceable lengths of 87 mm and 108 mm. In the EID-DECT group, two extrahepatic supplies confirmed by DSA were not identified preoperatively. Because the number of extrahepatic blood-supply cases was small, these observations are presented descriptively.

Regarding radiation exposure, the PCCT group showed lower CTDIvol and DLP than the EID-DECT group: 50.76 ± 9.69 versus 89.71 ± 19.51 mGy for CTDIvol, and 1206.87 ± 288.70 versus 1927.87 ± 291.99 mGy·cm for DLP (both *p* < 0.001).

### 3.3. Interventional Outcomes

In the dynamic-fusion PCCT group and the EID-DECT group, the number of angiographies before the first successful superselective catheterization was 1.20 ± 0.41 and 1.60 ± 0.51, respectively; the total number of angiographies was 5.00 ± 1.46 and 6.20 ± 1.66, respectively. The CBCT utilization rate, fluoroscopy time, and intraoperative contrast dose for both groups are presented in Table 5.

## 4. Discussion

The primary finding of this study is that dynamic multi-phase fused PCCT not only improves preoperative visualization of culprit vessels in HCC but also enhances the completeness of feeding artery mapping and the ability to predict DSA pathways. Within the same patients, compared with the optimal single-phase PCCT, dynamic fused PCCT increases patient-level coverage of DSA-confirmed culprit vessels and shows advantages in constructing a comprehensive feeding artery map and predicting the first-choice DSA pathway. A comparison with the preset group of EID-DECT also indicates potential benefits. Importantly, this study’s evaluation goes beyond assessing vessel visibility: it examines whether preoperative CT can comprehensively depict tumor-feeding arteries and their continuous pathways, corresponding to the culprit vessels ultimately confirmed by DSA and the actual interventional routes. Thus, the value of dynamic multi-phase fused PCCT may extend from improved vessel visualization to enabling pre-TACE construction of feeding artery maps and planning of interventional pathways.

DSA and selective or superselective angiography remain essential for identifying the culprit vessels of HCC during TACE. In this study, the final culprit vessels were determined by integrating DSA, selective/superselective angiography, CBCT (if available), and the actual embolization records, and the preoperative predicted path was evaluated against the actual successful superselective path. Previous work has shown that C-arm CT or CBCT can complement two-dimensional DSA in recognizing tumor-feeding arteries and enhance visualization of small culprit vessels [4,15,16]. Building on those findings, we expanded the preoperative CT assessment beyond general image-quality metrics such as vascular clarity and branch display to include culprit-vessel coverage, map integrity, and path prediction, thereby aligning imaging evaluation more closely with the actual TACE procedural pathway.

Previous Angio-CT and CBCT studies have shown that multi-phase and three-dimensional vascular information improves identification of culprit vessels and facilitates interventional navigation. However, these data are typically acquired only during procedures. Ohta et al. combined early and late arterial-phase Angio-CT images when needed and achieved a 98.0% identification rate for HCC culprit vessels, with successful subsegmental or more distal cannulation in all cases [8]. Takada et al. generated a three-dimensional feeding artery map from Angio-MDCT that enabled rapid identification of tumor-feeding arteries and guided TACE catheter selection [7]. Minamiguchi et al. used CT during aortography to map hepatic tumor feeders and similarly showed that three-dimensional vascular information aids catheter-path planning [9]. Dual-phase CBCT navigation has been reported to enhance tumor and feeding artery visualization, reduce the number of DSA runs and fluoroscopy time, and increase the rate of superselective embolization [6]. A systematic review also found that CBCT-based automatic feeding artery detection has high sensitivity for hepatic tumor feeders [5,17]. Together, these studies suggest that integrating multiple arterial time points with three-dimensional vascular data can reduce reliance on repeated two-dimensional DSA searches for culprit vessels during TACE.

Compared with the interventional indoor navigation techniques described above, this study specifically examines whether a usable atlas of responsible vessels with path-planning value can be obtained prior to TACE. Prior work combining CBCT with automatic responsible-vessel detection software has shown that three-dimensional blood-supply information can both reveal small intrahepatic or extrahepatic feeding arteries and potentially improve selective TACE implementation [17,18]. Here, we use preoperative intravenous enhanced dynamic PCCT to integrate continuous arterial-phase information and broaden the evaluation endpoints beyond simple vessel depiction to include: the coverage rate of responsible vessels confirmed by DSA, the rate of complete atlases, prediction accuracy for the first-choice path, and intervention-related procedural metrics. We do not propose replacing DSA with preoperative PCCT; rather, we ask whether PCCT can provide more complete information about responsible vessels and their continuous trajectories before the procedure, thereby reducing exploratory maneuvers to locate and confirm responsible vessels during DSA.

The primary benefit of dynamic-fusion PCCT over the optimal single-phase PCCT stems from integrating complementary information across distinct arterial phases to improve continuous feeding artery tracking, rather than merely increasing vascular clarity. The early arterial phase yields strong arterial enhancement with minimal venous contamination, whereas later phases better depict distal branches and tumor-terminal perfusion. No single time point can simultaneously optimize visualization of proximal arteries, distal-branch continuity, and tumor enhancement. Prior dual-arterial-phase CT work has confirmed complementary value of different arterial timings for hypervascular HCC [10], and the Angio-CT study by Ohta et al. supports multi-phase fusion for feeding artery detection [8]. Our vascular-level paired analysis corroborates these findings: dynamic-fusion PCCT achieved higher maximum trackable branch levels and improved vascular continuity, with only modest differences in vascular clarity.

The optimization cohort assessed small-caliber normal hepatic artery branches in patients with cirrhosis without HCC, where dynamic fusion conferred more prominent gains in distal-vessel depiction. By contrast, HCC culprit vessels are frequently enlarged and hyperenhanced by tumor perfusion, already showing good conspicuity on single-phase images. Accordingly, incremental gains from dynamic fusion in HCC largely reflect better vascular continuity and distal trackability instead of superior raw clarity. Of note, both PCCT protocols attained generally high subjective scores for vascular clarity and continuity, with most ratings near the upper scale limit, suggesting a potential ceiling effect.

The technical characteristics of PCCT underpin multi-phase vascular information integration, yet improvements in feeding artery mapping cannot be ascribed merely to higher SNR and CNR. By directly counting X-ray photons, PCCT reduces electronic noise and delivers high spatial resolution alongside native multi-energy imaging capacity [11,12,13,19,20]. A within-patient abdominal arterial-phase CT comparison by Graafen et al. demonstrated that low-keV PCCT virtual monoenergetic images enhance contrast and overall image quality for hepatic vessels and hypervascular liver lesions [21]. Other abdominal vascular investigations reported extended trackable arterial lengths and superior distal-branch visualization with PCCT [22]. Wang et al. noted that 0.2 mm ultra-high-resolution PCCT yielded better depiction of small visceral arteries than thicker-slice reconstructions despite sub-maximal objective SNR and CNR [14]. Recent ultra-high-resolution abdominal vascular studies further highlight that PCCT’s vascular benefits cannot be quantified by conventional SNR/CNR alone, given trade-offs between spatial resolution, reconstruction algorithms, and low-keV imaging [23,24]. Thus, the improved feeding artery maps observed herein most likely arise from the combined effects of high spatial resolution, thin-slice acquisition, multi-energy reconstruction, and multi-phase fusion, instead of enhancement in any single objective image-quality metric.

Between-group comparison against EID-DECT reveals potential strengths of dynamic-fusion PCCT for preoperative feeding artery mapping, although these findings require cautious interpretation owing to the small sample size and non-paired design. Prior intra-patient abdominal studies demonstrated that PCCT outperforms third-generation dual-source EID-CT in low-keV imaging, CNR, and subjective image quality of abdominal parenchyma and vessels at comparable radiation doses [21,25]. Chest and abdominal comparative studies also support PCCT’s capability to maintain or elevate image quality while lowering radiation exposure [21,24,26]. Importantly, our comparison evaluates two full scanning-postprocessing workflows rather than detector hardware in isolation. Dynamic-fusion PCCT displayed favorable outcomes for patient-level feeding artery coverage, complete atlas generation, path prediction, and detection of unexpected intraoperative culprit vessels, though inter-group differences in patient-level coverage did not reach statistical significance. The PCCT group also showed a non-significant trend toward fewer pre-superselective and total angiographic runs. Given the absence of intra-patient matching, these results should be interpreted as potential workflow-related merits of dynamic-fusion PCCT for preoperative mapping and path-planning, rather than definitive hardware superiority of PCCT over EID-DECT.

Identification of extrahepatic collateral supply constitutes another critical clinical application for comprehensive preoperative feeding artery mapping. HCC may recruit extrahepatic feeders including the inferior phrenic, adrenal, intercostal, and left gastric arteries, especially for large, subcapsular, hepatic-apical lesions or those with prior local therapy [27,28]. These vessels demonstrate considerable anatomic variation and frequently overlap with adjacent soft-tissue structures such as the diaphragm and gastric wall. Preoperative failure to detect them may result in unaddressed tumor perfusion or increase intraoperative vessel-hunting complexity. Prior CBCT and automated feeding artery-detection studies have highlighted the supplementary value of three-dimensional imaging for identifying extrahepatic supply [17,18]. In our cohort, DSA confirmed two extrahepatic feeders in each of the PCCT and EID-DECT groups. Dynamic-fusion PCCT preoperatively detected and achieved relatively long tracking of these vessels, whereas optimal single-phase PCCT and EID-DECT did not. Though this observation is consistent with improved long-range vascular tracking via multi-phase integration, the very small number of positive vessels renders it exploratory; definitive inter-protocol differences in extrahepatic-supply detection cannot be concluded.

This work differs from earlier PCCT and TACE-navigation investigations by translating PCCT technical strengths into preoperative assessment of continuous feeding artery atlases and interventional pathways. Most existing abdominal PCCT publications concentrate on technical and image-quality metrics: spatial resolution, reconstruction kernels, low-keV virtual monoenergetic images, noise, CNR, and radiation dose [14,19,20,21,22,23,24]. Only recently have studies begun evaluating whether these technical gains translate into tangible clinical-task performance [19]. Meanwhile, conventional HCC feeding artery navigation has largely depended on intraprocedural Angio-CT, CBCT, and automated feeder-detection tools [5,6,7,8,9,17,18]. We constructed preoperative continuous feeding artery atlases spanning vascular origins, branching chains, and tumor peripheries using dynamic PCCT, with validation against DSA-confirmed feeders and real-world procedural pathways. This shifts the evaluation focus from mere vascular conspicuity toward whether preoperative atlases can support interventional path-planning. This approach is not intended to replace DSA; instead, it delivers richer pre-procedural vascular information to minimize intraoperative exploratory maneuvers and facilitate superselective catheterization for optimized TACE workflows.

This study has several limitations. First, the sample size is small; post hoc power estimation based on the observed effect size suggested that the present cohort achieved moderate statistical power for the primary within-patient comparison (dynamic-fusion PCCT versus optimal single-phase PCCT). However, statistical power was limited for inter-group comparison between dynamic-fusion PCCT and EID-DECT, especially for infrequent outcomes such as extrahepatic feeding artery detection. Accordingly, findings from the present cohort should be regarded as hypothesis-generating rather than definitive clinical evidence. Second, comparisons between PCCT and EID-DECT were inter-patient rather than intra-patient paired analyses, so inter-individual variability and potential confounders could bias the results. Third, interventional operators may modify intraoperative angiographic and vessel-hunting strategies after reviewing pre-procedural dynamic-fusion maps, creating workflow-related bias for our composite reference standard. Finally, continuous multi-arterial-phase acquisition generates large datasets and increases post-processing demands. Despite its advantages in spatial resolution and multi-energy reconstruction, real-world PCCT implementation requires careful trade-offs between data handling, radiation dose, and clinical workflow efficiency [29]. Therefore, the clinical value of dynamic multi-phase fusion PCCT needs further multicenter validation with larger cohorts to assess its effects on angiographic frequency, fluoroscopy time, contrast agent usage, and overall interventional performance.

## 5. Conclusions

Dynamic multi-phase fusion PCCT expands coverage of responsible blood vessels before TACE in patients with HCC compared with the optimal single-phase PCCT, and it enhances construction of a complete vascular atlas and prediction of the first-choice DSA pathway; it also shows potential advantages over EID-DECT. This technique therefore promises more comprehensive imaging for identifying feeding arteries and planning interventional routes before TACE, with the potential to reduce intraoperative exploration and streamline the interventional workflow. Notably, there was no statistically significant inter-group difference in patient-level feeding artery coverage between dynamic-fusion PCCT and EID-DECT. Real-world barriers including limited scanner access, high costs, and added patient radiation burden should also be acknowledged. Cost-effectiveness analyses are required before routine clinical adoption of this multi-phase fused PCCT workflow for pre-TACE mapping.

## Figures and Tables

**Table 1:** Baseline characteristics of patients.

Characteristic	PCCT (*n* = 15)	EID-DECT (*n* = 15)	*p* Value
Age (years),	60.8 ± 10.2	61.4 ± 9.8	0.888
Sex (male/female), *n*	12/3	12/3	1.000
Etiology of cirrhosis, *n* (%)			0.550
HBV	13 (86.7)	14 (93.3)	
Alcoholic	1 (6.7)	0 (0)	
Other/Unknown	1 (6.7)	1 (6.7)	
Child–Pugh class, *n* (%)			0.288
A	7 (46.7)	10 (66.7)	
B	8 (53.3)	5 (33.3)	
BCLC stage, *n* (%)			0.198
A	1 (6.7)	1 (6.7)	
B	10 (66.7)	6 (40.0)	
First TACE for target lesion, *n* (%)			0.345
Yes	4 (26.7)	6 (40.0)	
No	11 (73.3)	9 (60.0)	
Number of lesions,	2.0 ± 1.4	3.1 ± 1.7	0.052
Maximum lesion diameter (mm),	38.6 ± 12.5	39.2 ± 11.8	0.982
Liver segment of target lesion, *n* (%)			0.870
S1–S8 (single segment)	12 (80.0)	11 (73.3)	
Multi-segments	3 (20.0)	4 (26.7)	
High-risk location for extrahepatic supply, *n* (%)			0.345
Yes	2 (13.3)	4 (26.7)	
No	13 (86.7)	11 (73.3)	
DSA-confirmed feeding arteries, M (Q1, Q3)	2 (1, 3)	2 (1, 3)	0.830

Note. PCCT = photon-counting computed tomography; EID-DECT = energy-integrating-detector dual-energy computed tomography; HBV = hepatitis B virus; BCLC = Barcelona Clinic Liver Cancer; Q1 = first quartile; Q3 = third quartile.

**Table 2:** Paired comparison between dynamic-fusion PCCT and optimal single-phase PCCT.

Outcome	Dynamic Fused PCCT	Optimal Single-Phase PCCT	95% CI	*p* Value
DSA-confirmed responsible-vessel coverage rate,	97.78 ± 8.61	88.33 ± 15.37	9.44 (1.82–17.06)	0.016
complete feeding-vessel mapping rate, *n* (%)	14 (93.3)	8 (53.3)		0.039
first-choice DSA-path-prediction accuracy	96.67 ± 11.18	80.00 ± 24.49	16.67 (2.15–31.19)	0.032
The number of intraoperatively missed feeding vessels,	0.07 ± 0.26	0.40 ± 0.61	−0.33 (−0.58–0.09)	0.021
Tumor SNR	14.85 ± 3.22	12.36 ± 2.89	2.49 (0.58–4.40)	0.012
Tumor CNR	6.82 ± 2.15	5.21 ± 1.98	1.61 (0.35–2.87)	0.014

Note. SNR = signal-to-noise ratio; CNR = contrast-to-noise ratio.

**Table 3:** Per-vessel paired Wilcoxon signed-rank test (dynamic-fusion PCCT vs. optimal single-phase PCCT, 36 paired vessels).

Parameter	Dynamic Fused PCCT	Optimal Single-Phase PCCT	Median Difference (Fusion—Single-Phase)	Z	*p*	Improved/ Decreased/Unchanged
Maximum traceable branch level	5 (5, 5)	5 (4, 5)	0 (0–1)	−2.808	0.005	14/0/22
Vascular clarity	5 (5, 5)	5 (5, 5)	0 (0–0)	−1.732	0.083	3/0/33
Vascular continuity	5 (5, 5)	5 (5, 5)	0 (0–0)	−2.000	0.046	4/0/32

Note. A total of 36 DSA-confirmed culprit vessels from 15 patients were included. Ceiling effect was observed for vascular clarity and continuity, as baseline scores were generally close to the maximum (5 points).

**Table 4:** Comparison between dynamic-fusion PCCT and EID-DECT.

Outcome	Dynamic Fused PCCT	EID-DECT	95% CI	*p*
DSA-confirmed responsible-vessel coverage rate,	97.78 ± 8.61	90.45 ± 12.31	7.33 (−1.01–15.67)	0.083
complete feeding-vessel mapping rate, *n* (%)	14 (93.3)	8 (53.3)		0.035
first-choice DSA-path prediction accuracy	96.67 ± 11.18	83.33 ± 18.44	13.34 (1.26–25.42)	0.032
The number of intraoperatively missed feeding vessels	0.07 ± 0.26	0.40 ± 0.51	−0.33 (−0.64–−0.02)	0.040

**Table 5:** DSA-related and interventional procedural outcomes.

Outcome	Dynamic Fused PCCT	EID-DECT	*p* Value
the number of angiographic runs prior to the first successful superselective catheterization	1.20 ± 0.41	1.60 ± 0.51	0.024
the total number of angiographic runs	5.00 ± 1.46	6.20 ± 1.66	0.039
CBCT utilization, *n* (%)	3 (20.0)	6 (40.0)	0.227
Fluoroscopy time (min),	9.75 ± 2.34	14.71 ± 3.02	<0.001
Intra-procedure contrast volume (mL)	130.0 ± 27.5	154.0 ± 26.9	0.018

## Data Availability

Raw clinical and imaging data cannot be made publicly available due to patient-related privacy and ethical restrictions. The de-identified datasets are available from the corresponding author upon reasonable request and with formal ethics approval.

## Abbreviations

HCC	Hepatocellular carcinoma
PCCT	Photon-counting computed tomography
EID-DECT	Energy-integrating-detector dual-energy computed tomography
DSA	Digital subtraction angiography
TACE	Transarterial chemoembolization
CBCT	Cone-beam computed tomography
CTA	CT angiography
SNR	Signal-to-noise ratio
CNR	Contrast-to-noise ratio
BCLC	Barcelona Clinic Liver Cancer
CTDIvol	Volume computed tomography dose index
DLP	Dose-length product
MIP	Maximum intensity projection
ROI	Region of interest
